# Supplementary figures and images for: Role of syndecan-1 in the interaction between dendritic cells and T cells
Source: PLoS One. 2020 Jul 23;15(7):e0230835. doi: 10.1371/journal.pone.0230835 (PMC7377417; doi:10.1371/journal.pone.0230835)

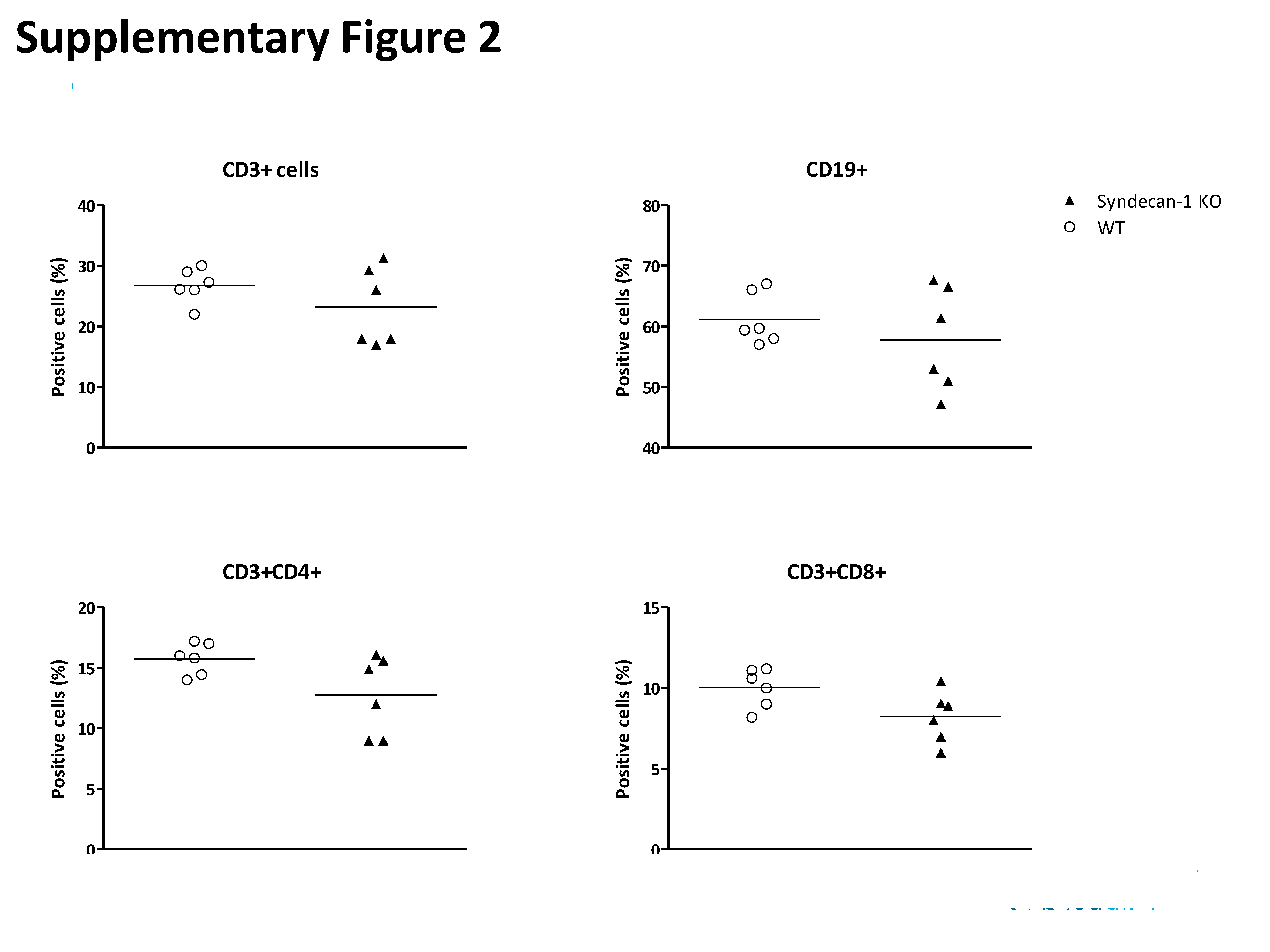

Supplement: S2 Fig — Splenocytes of Sdc-1 deficient and WT mice were analyzed for expression of CD3, CD4, CD8 and CD19 by flow cytometry. Experiments were replicated 5 times. Results are expresses as mean ± standard error of means. (TIF) [file pone.0230835.s002.tif]

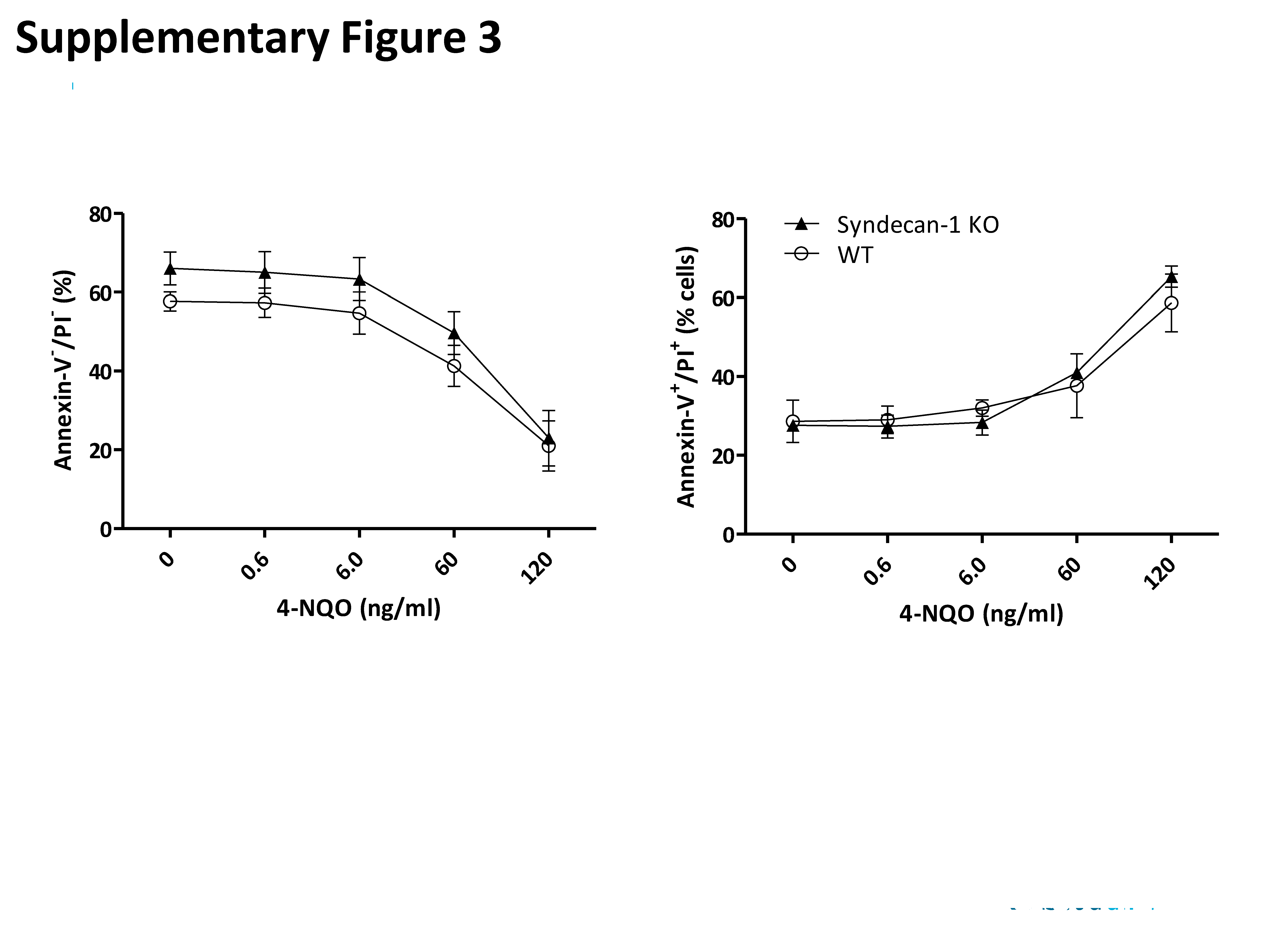

Supplement: S3 Fig — Sdc-1 deficient or WT splenocytes were incubated with low dose 4-nitroquinoline 1-oxide, stained with Annexin V–propidium iodide and analyzed by flow cytometry. Experiments were replicated 3 times. Results are expresses as mean ± standard error of means. (TIF) [file pone.0230835.s003.tif]
