# Supplementary material for: Role of syndecan-1 in the interaction between dendritic cells and T cells
Source: PLoS One. 2020 Jul 23;15(7):e0230835. doi: 10.1371/journal.pone.0230835 (PMC7377417; doi:10.1371/journal.pone.0230835)
Supplement: Attachment — Submitted filename: PONE-D-20-06732_Comments_Ibrahim SA.doc [file pone.0230835.s004.doc]

PONE-D-20-06732_Comments

Kouwenberg M et al., studied the interaction between dendritic cells (DC) and T- cells deciphering the role of Syndecan-1 in the context of graft survival in an allogeneic transplant model. The authors found that Syndecan-1 depletion did not affect cytokine production, expression of co-stimulatory molecules, or T cell stimulatory capacity of DC. Additionally, ConA activation of T-cells by ConA or DC induced Syndecan-1 expression associated with lower proliferative potential and IL-17 production. This study suggests a role for intracellular Sdc-1 in function of T cells, however, without beneficial effect for prolongation of allograft survival with a complete MHC mismatch. Overall, the study is well-designed and well-written. Only one note; a recent article should be mentioned as they are among the few that have shown the correlation between Syndecan-1 and T-cells (Saleh ME, et al., The Immunomodulatory Role of Tumor Syndecan-1 (CD138) on Ex Vivo Tumor Microenvironmental CD4+ T Cell Polarization in Inflammatory and Non-Inflammatory Breast Cancer Patients, PLoS One 2019 May 30;14(5):e0217550).

Sincerely,

***Sherif Ibrahim***
